# Retrospective Analysis of Histopathological Reports of Salivary Gland Pleomorphic Adenomas in Tanzania

**DOI:** 10.24248/eahrj.v8i2.781

**Published:** 2024-06-26

**Authors:** Jeremiah Robert Moshy, Karpal Singh Sohala, Fredrick M Sebasaza, Gemma Berege

**Affiliations:** a Muhimbili University of Health and Allied Sciences, Dar es Salaam, Tanzania; b Muhimbili National Hospital, Dar es Salaam, Tanzania

## Abstract

**Background:**

Pleomorphic adenoma (PA) is the most common benign tumor representing about 80% of all salivary gland tumors. Despite this, there is limited documentation of the demographic information and pattern of PA in Tanzania. This study retrospectively determines the demographic information and the pattern of presentation of pleomorphic adenomas of the salivary gland among patients managed at a tertiary hospital in Tanzania.

**Methods:**

This was a retrospective study of histological results of salivary gland pleomorphic adenoma diagnosed between 2016 and 2021. The information gathered included the age and sex of the patient and the anatomical location. Data analysis was done using Statistical Package for the Social Sciences version 27 computer program.

**Results:**

Out of 1824 reports of patients with oral and maxillofacial lesions retrieved from the archives of the department, 62 (3.4%) had the diagnosis of pleomorphic adenoma of the salivary glands. The patients' ages at diagnosis ranged from 7 to 72 years, with a mean age of 39.9 (SEM = 2.3) years. The male-to-female ratio of patients diagnosed with pleomorphic adenoma was 1:1. There were 31 (50%) cases of pleomorphic adenomas affecting major salivary glands. The palatal minor salivary glands were the most (n=31, 50%) affected followed by the parotid gland (n=16, 25.8%).

**Conclusion:**

Pleomorphic adenomas have no sex predilection, most of these lesions occur during the 3^rd^ to 5^th^ decade of life. The majority of pleomorphic adenomas occur in the palatal minor salivary glands.

## BACKGROUND

Salivary gland tissues are divided into two main types, the major salivary glands (parotid, submandibular, and sublingual glands) and the minor salivary glands which are present in the mucosal lining of aerodigestive track including sites such as the lips, gingiva, cheek, palate, tongue, oropharynx, paranasal sinuses and parapharyngeal space.^1^ The salivary glands are prone to various tumors that account for 1% to 4% of all neoplasms of the human body^2^ and of these, pleomorphic adenoma (PA) is the most common benign tumor representing about 80% of all salivary gland tumors.^3,4^ The palate is the most common intraoral site for PA, whilst the parotid is the most frequently affected major salivary gland.^2,3,5^

Generally, the PA tends to occur commonly in females between the fifth and seventh decades of life, and rarely in children and adolescents.^2,6^ Data from the Polish National Registry of benign salivary gland tumors revealed that the age range of patients diagnosed with PA was from 13 to 86 years, mean of 47.93±14.93 years and 64.8% of the patients were women.^6^ A study from Taiwan^5^ reporting on the palatal PA, showed a marked female predilection, and the patients were in their 3rd to 8th decades of life with a mean age of 47 years.

Several previous studies from Tanzania have reported that pleomorphic adenoma is among the top five most diagnosed benign lesions,^7–9^ yet still, most of the information presented to date has been general and not specific to the pleomorphic adenoma. Thus, there is limited documentation of the demographic information and pattern of PA in Tanzania. Considering the paramount significance of having documented baseline information regarding PA in Tanzania, this study aims to determine the demographic information and the pattern of presentation of pleomorphic adenomas of the salivary gland among patients managed at a tertiary hospital in Tanzania. Results of this study provides important information that will aid cliniciand in bringing up the definite characteristics and distribution of the salivary gland pleomorphic adenoma in Tanzania and thus the development of locally tailored interventions for its management.

## METHODOLOGY

### Study Design

This was a retrospective study of histopathologic reports of biopsied orofacial lesions of patients attended at the Muhimbili National Hospital (MNH), Tanzania. All histopathological reports of oral and maxillofacial lesions obtained from 2^nd^ January 2016 to 31^st^ December 2021 were obtained. Only those reports that had a final diagnosis indicating a pleomorphic adenoma (PA) were included. The Reports with inconclusive diagnoses or without a final diagnosis were excluded.

### Study Area

This study was carried out at the MNH. The hospitsl has a well-established oral and maxillofacial center in the country, and being a tertiary health facility, it receives patients with various oral and maxillofacial conditions including pleomorphic adenomas from all over the country.

### Data Collection

A data collection form was used to extract information from the histopathology reports. The information captured included the age and gender of the patient, the location of the lesion, histological diagnosis, and histological identification number. In a case where a single patient had more than one result, as one for pre-surgery incisional biopsy and another for post-surgical excision of the lesion, the post-op result was included.

### Data Analysis

The data obtained from this study were coded and analyzed using Statistical Package for Social Sciences software (SPSS) for Windows (version 27, Armonk, New York: IBM Corp). The continuous variables were presented in the form of the mean and percentages while the categorical variables were presented in the form of tables. For descriptive analysis, the patients' age was grouped into 4 groups: pediatrics (<18 years), young adults (18 – 39 years), middle-aged adults (40 – 59 years), and older adults (60+ years). For regressional analysis, the age was dichotomized into ≤40 years and >40 years. The location of the lesion was grouped into those involving major salivary glands (parotid, submandibular, and sublingual) and minor salivary glands.

To assess the relationship between sociodemographic characteristics and the nature of the lesion either Oneway Analysis of Variance (ANOVA) or chi-square tests were used. The *P* value of less than 0.05 was selected for statistical significance. Multivariate logistic regression models were used to assess the degree to which the age and sex of patients were associated with the occurrence of the lesion.

### Ethical Approval

Ethical clearance was sought from the MUHAS research and ethics committee (DA.25/111/01B/208), and permission to conduct the study was obtained from the appropriate authorities of the Department of Oral and Maxillofacial Surgery. Confidentiality and privacy of patients' information were observed during the entire process of data collection.

## RESULTS

A total of 1824 reports of patients with oral and maxillofacial lesions were retrieved from the archives of the department. Of these, 62 (3.4%) had the diagnosis of pleomorphic adenoma. The patients' ages at diagnosis ranged from 7 to 72 years, with a mean age of 39.89 (SEM=2.29) years. The 18-39 years age group was more affected (N=24, 38.7%). There was an equal distribution of participants by sex (M:F=1:1).

**Figure 1.**
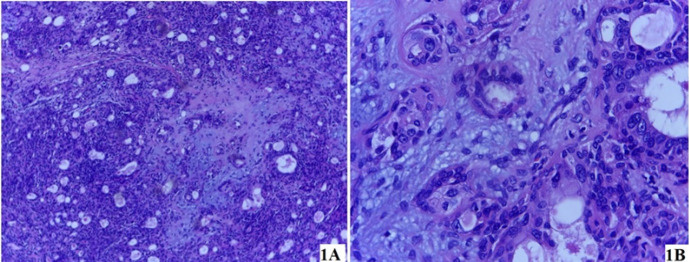
Photomicrograph (Haematoxylin and Eosin Stain) of the Pleomorphic Adenoma (a) Section of tissue showing a benign tumor with epithelial and myoepithelial components in myxoid stroma, with areas of focal intervening fibrotic stroma (magnification x10). (b) Section of tissue showing mainly epithelial component (glandular structure) in chondromyxoid stroma (magnification x40).

**Figure 2.**
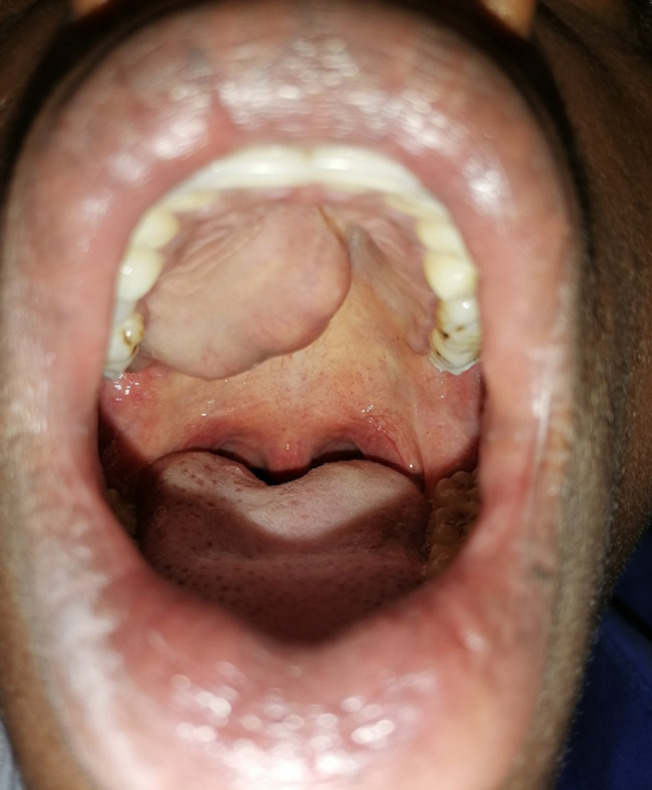
Clinical Picture of Pleomorphic Adenoma of the Palate

The major and minor salivary glands were equally (1:1) affected by pleomorphic adenomas (Figure 1). The palatal minor salivary glands were the most (n=31, 50%) affected (Figure 2) followed by the parotid gland (n=16, 25.8%). The palatal minor salivary glands were commonly affected among middle-aged adults, while in young adults, the parotid gland and submandibular salivary glands were frequently affected sites. Except for the pleomorphic adenomas of the palatal minor salivary gland which were frequent among the females, pleomorphic adenomas of the rest of the salivary glands were more found in males (Table 1).

**Table 1.** Distribution of Patients with Salivary Gland Pleomorphic Adenoma Patients According Age Group, Sex and Tumour Site

Age and sex of the participants	Palatal (n=31)	Salivary gland affected Parotid (n=16)	Submandibular (n=13)	Sublingual (n=2)
Age Groups (years)				
<18	2 (6.5%)	4 (25%)	1 (7.7%)	-
18-39	11 (35.5%)	7 (43.8%)	5 (38.5%)	1 (50.0%)
40-59	14 (45.2%)	2 (12.5%)	3 (23.1%)	1 (50.0%)
60+	4 (12.9%)	3 (18.8%)	4 (30.8%)	-
Mean age	42	34	43	43
Sex				
Female	18 (58.1%)	7 (43.8%)	5 (38.5%)	1 (50.0%)
Male	13 (41.9%)	9 (56.3%)	8 (61.5%)	1 (50.0%)

The occurrence of pleomorphic adenoma in the submandibular salivary gland and the palatal minor salivary glands was not determined by either sex or age group of patients. However, for the pleomorphic adenoma of the parotid glands, the age group was a determinant factor (*P*=.032)., whereby the likelihood of pleomorphic adenoma occurring in the parotid gland decreased by 74% in patients aged above 40 years (Table 2).

**Table 2.** Binary Association of Pleomorphic Adenomas with Age and Sex

Age groups of the participants	Location of Pleomorphic Adenoma		
	Palatal UOR (95%CI)	Parotid UOR (95%CI)	Submandibular UOR (95%CI)
Age Groups			
≤40 years	Ref	Ref	Ref
>40 years	2.19 (0.79-6.05)	0.26 (0.72-0.92)	1.32 (0.39-1.93)
*P value*	.130	.036	.658
Sex			
Male	Ref	Ref	Ref
Female	1.92 (0.7-5.26)	0.71 (0.23-2.24)	0.55 (0.16-1.93)
*P value*	.21	.562	.353

## DISCUSSION

Due to their vast array of biological behavior and morphological spectrum, salivary gland tumors often pose diagnostic challenges and consequently in their treatment even in the most equipped centers in the world.^2,10^ The aggressive biological nature of benign tumors as demonstrated by a high rate of recurrence and repeated surgical failures in pleomorphic adenoma may lead to misdiagnosis.^11^ This is more so in the African continent, where diagnostic resources are limited and thus a need for a thoughtful management plan.^10^ Taking this into account, coupled with the importance of epidemiological studies in bringing up the definite characteristics and distribution of the disease in a specific environment and thus the development of tailored interventions,^12,13^ it is necessary to have local epidemiological information regarding pleomorphic adenoma (PA) in Tanzania.

The incidence of orofacial lesions varies from one geographical location to another^14^. In the current audit, PA constituted 3.4% of the biopsied orofacial lesions. This was higher than findings from Kenya,^15^ Nigeria,^16,17^ and Bangladesh^18^ but similar to findings from Uganda^19^ and another report from Nigeria.^20^ Among the reasons for this disparity between countries may be differences in study methodology and the capacity of the health facility where the study was carried out.

According to the data in the literature, pleomorphic adenoma of the salivary glands has a predilection for females,^2,3,6^ to the contrary, the results from this study showed no gender predilection similar to findings from Ghana.^21^ Though in this series, the PA of the palate was more common in females, similar to reports from elsewhere,^5,22^ the major salivary glands (parotid and submandibular) had slight male preponderance, results which are contrary to reports in the literature^3,23,24^ but similar to report for South Africa.^25^ The difference noted in this study calls for a multicentric study that specifically looks into salivary glands among Africans considering the paucity of epidemiological studies on salivary gland pleomorphic adenomas from Africa, considering that we rely on information from a vast amount of literature from the Western population.

The data from the current study portrays that the patients' ages at diagnosis ranged from 7 to 72 years, with a mean age of 39.89 years, and peak incidence around the 3^rd^ to 5^th^ decade. Such findings are consistent with the reports in the literature.^3,6,26^ Though there may be some variations in mean ages in reports from different countries,^3,24,27^ which may be attributed to racial differences. Considering the wide age range of occurrence, it is worth considering PA as a differential diagnosis of benign lesions affecting the salivary gland at any age.

In the present study, it was noted that minor salivary glands of the palate were most affected by PA followed by the parotid gland. This was similar to the findings by Lopes et al.^3^ but contrary to several other reports,^1,21,28,29^ yet still Vuhahula^11^ found the submandibular gland was the most affected. Such peculiar findings of a relatively high incidence of PA in the palatal salivary gland compared to the parotid gland is worth to be commented bearing in mind it is different in comparison to the western series. Although the explanation for this is not clear, one of the many reasons could be the difference in various centers where the studies are carried out.

The occurrence of pleomorphic adenoma of salivary glands was not determined by either sex or age group of patients except for parotid glands, where the likelihood of pleomorphic adenoma occurring in the parotid gland decreased by 74% in patients aged above 40 years. Though the exact reason is not clear, it may be hypothesized that it is partially related to relative decreases in the acinar cell component of the salivary glands and/or alterations in receptor function and signal transduction that occurs with aging.^30^

This study had a few limitations which are worth acknowledging. Despite our institute serving a majority of Tanzanian patients with orofacial tumors and tumor like lesions, some cases are treated elsewhere. Hence it may be suspected that not all PAs were included. Secondly, some reports had been excluded from the study due to missing information regarding the site of lesion. Nevertheless, this is a study of the kind in Tanzania, as it provides valuable information on the pleomorphic adenomas of salivary glands with respect to age, gender, and anatomical location.

## CONCLUSION

The findings of the current study indicates that pleomorphic adenomas of the salivary gland have no sex predilection, with the peak age of occurrence being between the third and 5^th^ decade of life. The palatal minor salivary glands are the most affected followed by the parotid gland. It is recommended that clinicians to consider PA as a differential diagnosis of benign lesions affecting the salivary gland at any age.
